# Heat Bath in a Quantum Circuit

**DOI:** 10.3390/e26050429

**Published:** 2024-05-17

**Authors:** Jukka P. Pekola, Bayan Karimi

**Affiliations:** 1 Pico Group, QTF Centre of Excellence, Department of Applied Physics, Aalto University, P.O. Box 15100, FI-00076 Aalto, Finland; bayan.karimi@aalto.fi; 2 QTF Centre of Excellence, Department of Physics, Faculty of Science, University of Helsinki, FI-00014 Helsinki, Finland

**Keywords:** heat bath, superconducting circuit, resonator, Josephson array, resistor

## Abstract

We discuss the concept and realization of a heat bath in solid state quantum systems. We demonstrate that, unlike a true resistor, a finite one-dimensional Josephson junction array or analogously a transmission line with non-vanishing frequency spacing, commonly considered as a reservoir of a quantum circuit, does not strictly qualify as a Caldeira–Leggett type dissipative environment. We then consider a set of quantum two-level systems as a bath, which can be realized as a collection of qubits. We show that only a dense and wide distribution of energies of the two-level systems can secure long Poincare recurrence times characteristic of a proper heat bath. An alternative for this bath is a collection of harmonic oscillators, for instance, in the form of superconducting resonators.

## 1. Introduction

The question of thermalization in closed quantum systems and the nature of thermal reservoirs are topics of considerable interest [1,2,3,4,5,6,7]. However, experimental realizations, in particular, in the solid-state domain are largely missing [4,8]. In this paper, we compare different types of reservoirs that can be realized in the context of superconducting quantum circuits. An ideal heat bath is a resistor [9,10,11,12,13,14,15], which can be realized in a straightforward way. However, mainly because of the compatibility of the fabrication processes, the circuit QED community typically prefers to mimic resistors or simply to produce high-impedance environments by arrays of Josephson junctions or superconducting cavities [16,17,18,19,20,21,22,23,24]. The advantages of a physical resistor in the form of a metal film are that it has a truly gapless and smooth absorption spectrum, and on the practical side its temperature can be probed by a standard thermometer [25]. A one-dimensional Josephson junction array, on the contrary, although acting as a high impedance environment [26,27], presents well-defined resonances in its absorption spectrum up to the plasma frequency and purely capacitive behavior above it and cannot thus be considered rigorously as a resistor. Experiments on multimode cavities support our conclusion, as they exhibit periodic recoveries of the qubit coupled to them [28]. In order to realize a Caldeira–Leggett type true reservoir [29,30] out of superconducting elements, we propose an ensemble of qubits or LC-resonators with a distribution of energies among them.

## 2. Basic Properties of LC Resonators and Josephson Arrays

**Figure 1:**
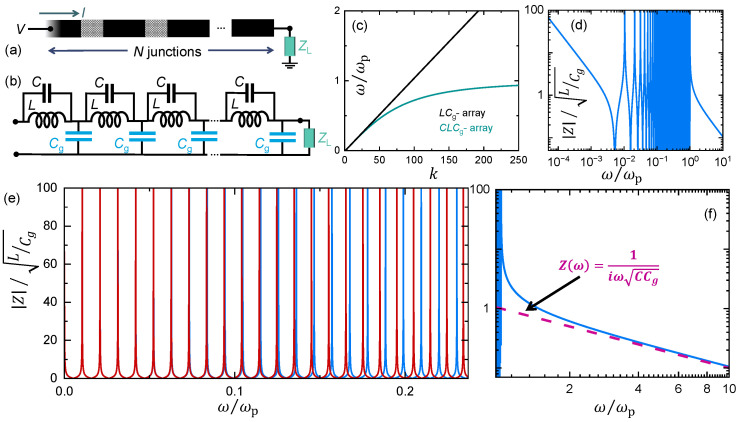
Basic properties of a one-dimensional Josephson junction array. (**a**) An array with *N* junctions, terminated by impedance $Z_{L}$. The current is *I*, and the voltage is *V*. Junctions can be replaced by superconducting interference devices (SQUIDs) acting as tunable junctions. (**b**) An equivalent circuit for a uniform array with junctions linearized as inductors *L*. The junction capacitance is *C*, and the stray “ground” capacitance of each island is $C_{g}$. (**c**) The dispersion relation for modes in the array for two cases, C=0 (black line, $LC_{g}$) and $C=100C_{g}$ (green line, $CLC_{g}$), for an array with N=3000. Here, we assume an open ended array ($Z_{L}=\infty$). The (angular) frequencies are scaled by the plasma frequency $\omega_{p}=1/\sqrt{LC}$ of each junction. (**d**) The modulus of the impedance of the $CLC_{g}$ array as a function of the frequency and (**e**) a zoom out of it for lower frequencies (red line), together with that of the linear $LC_{g}$ array as well (blue line). (**f**) At frequencies $\omega \gg \omega_{p}$, the $CLC_{g}$ array behaves as a capacitor with effective capacitance $\sqrt{CC_{g}}$.

We start with an elementary classical analysis of a one-dimensional Josephson junction array (see Figure 1a), which can be presented in a linearized form by a chain of parallel LC elements for the junctions, with a ground capacitance $C_{g}$ between two of them, as in Figure 1b. Assuming a long array, we can write for voltage V(k) on island *k* and current I(k), through the corresponding junction,

(1) $$\begin{array}{ccc} & & \partial V\left(k\right)/\partial k+Z_{LC}I\left(k\right)=0\\ & & i\omega C_{g}V\left(k\right)+\partial I\left(k\right)/\partial k=0. \end{array}$$

Here, $Z_{LC}=-iZ_{\infty }/\left(\omega /\omega_{p}-\omega_{p}/\omega \right)$ with $Z_{\infty }=\sqrt{L/C}$, ω is the angular frequency of driving, $\omega_{p}=1/\sqrt{LC}$ is the plasma frequency of the junction, and I(k) is the current through the *k*:th junction. One can solve these equations with different terminations of the array. One finds the dispersion relation of the angular frequencies $\omega_{n}$ for infinite impedance at

(2) $$\omega_{n}=\omega_{n,0}/\sqrt{1+{\left(\omega_{n,0}/\omega_{p}\right)}^{2}},$$

where, for an array of *N* junctions, $\omega_{n,0}=\left(n-1/2\right)\pi /\left(N\sqrt{LC_{g}}\right)$ for a shorted termination, and $\omega_{n,0}=n\pi /\left(N\sqrt{LC_{g}}\right)$ for an open line [20] (These frequencies practically coincide with those obtained by exact diagonalization of an array of arbitrary length and those from numerical results for n≪N, which is the usual regime). This is the functional dependence of the dispersion relation used in fitting the data, e.g., in Refs. [22,24], and it is depicted in Figure 1c for two different values of $C/C_{g}$, one for the pure LC transmission line $C/C_{g}=0$ and the other for $C/C_{g}=100$. Figure 1d–f shows the modulus of the frequency dependent impedance of an array calculated numerically for $C/C_{g}=100$. We conclude that such an array can hardly be considered to be a resistor. The resonant absorption at frequencies corresponding to Equation (2) is presented in experiments as well [22,24]. At frequencies above $\omega_{p}$, there are no more modes, and the impedance is purely capacitive, with impedance $Z\left(\omega \right)={\left(i\omega \sqrt{CC_{g}}\right)}^{-1}$ asymptotically at high frequencies (see Figure 1f).

## 3. LC Resonators and Josephson Arrays as Environment: Model

We next analyze the energy exchange between the system (here, a qubit) and a reservoir to assess whether the latter qualifies as a thermal bath. In general, an ideal array presents a reactive element that cannot dissipate the energy. Such a conclusion can be drawn for instance by analyzing the population of a qubit coupled to the array. To be concrete, we follow the model in Refs. [28,31] and consider a qubit with energy ℏΩ coupled to a bath of *N* states, with the energy of the *j*:th one equal to $\hbar \omega_{j}$. The Hamiltonian of the whole system and bath is given by

(3) $$\hat{H}=\hbar \Omega {\hat{a}}^{†}\hat{a}+\sum_{i=1}^{N}\hbar \omega_{i}{\hat{b}}_{i}^{†}{\hat{b}}_{i}+\sum_{i=1}^{N}\gamma_{i}\left({\hat{a}}^{†}{\hat{b}}_{i}+\hat{a}{\hat{b}}_{i}^{†}\right),$$

where $\hat{a}=\left|g\rangle \langle e\right|$ for the qubit with eigenstates |g〉 (ground) and |e〉 (excited), and ${\hat{b}}_{i}^{†}\;\left({\hat{b}}_{i}\right)$ is the creation (annihilation) operator of the environment modes. The non-interacting Hamiltonian is ${\hat{H}}_{0}=\hbar \Omega {\hat{a}}^{†}\hat{a}+\sum_{i=1}^{N}\hbar \omega_{i}{\hat{b}}_{i}^{†}{\hat{b}}_{i}$. The parameters $\gamma_{i}$ represent the coupling of the qubit with each state in the environment for the perturbation, which reads in the interaction picture with respect to ${\hat{H}}_{0}$ as

(4) $$\begin{array}{c} {\hat{V}}_{I}\left(t\right)=\sum_{i=1}^{N}\gamma_{i}\left({\hat{a}}^{†}{\hat{b}}_{i}e^{i(\Omega -\omega_{i})t}+\hat{a}{\hat{b}}_{i}^{†}e^{-i(\Omega -\omega_{i})t}\right). \end{array}$$

The basis that we use is formed of the states of the system and environment as $\{|0\rangle =|1000\ldots 0\rangle ,\;|1\rangle =|0100\ldots 0\rangle ,\ldots ,|i\rangle =|0\;0\ldots 1^{\left(i:\mathrm{th}\right)}\ldots 0\rangle \}$, where the first entrance refers to the qubit and from the second on to each of the *N* states in the bath. In what follows, we apply this model to both a multimode cavity and spins as environment. We choose the initial state of the whole system (qubit and environment) as $|\psi_{I}\left(0\right)\rangle \equiv |0\rangle$. This corresponds to the ground state of the environment (zero temperature, T=0) but with the qubit excited. The assumption of such a vacuum initial state is justified because, in typical experiments on superconducting qubits, the energy of the qubit is of the order of 1 K, whereas the temperature of the experiment is about 0.01 K. Since, especially in the weak coupling case, the qubit interacts mainly with degenerate states, the assumption of no excitations initially is a good one. We solve the Schrödinger equation $i\hbar \partial_{t}|\psi_{I}\left(t\right)\rangle ={\hat{V}}_{I}\left(t\right)|\psi_{I}\left(t\right)\rangle$ in the interaction picture to find the time evolution of the state of the whole system, $|\psi_{I}\left(t\right)\rangle =\sum_{i=0}^{N}C_{i}\left(t\right)|i\rangle$. In the given basis, the amplitudes $C_{i}\left(t\right)$ are then governed by

(5) $$\begin{array}{ccc} & & i\hbar {\dot{C}}_{0}=\sum_{j=1}^{N}\gamma_{j}e^{i(\Omega -\omega_{j})t}C_{j}\\ & & i\hbar {\dot{C}}_{j}=\gamma_{j}e^{-i(\Omega -\omega_{j})t}C_{0}. \end{array}$$

With the initial conditions $C_{0}\left(0\right)=1$ and $C_{j}\left(0\right)=0$ for j=1,…,N, i.e., with state |ψ(0)〉=|0〉, we find

(6) $$\begin{array}{ccc} & & C_{0}\left(t\right)=1-\frac{i}{\hbar }\sum_{j=1}^{N}\gamma_{j}\int_{0}^{t}dt^{\prime }e^{i\left(\Omega -\omega_{j}\right)t^{\prime }}C_{j}\left(t^{\prime }\right)\\ & & C_{j}\left(t\right)=-\frac{i}{\hbar }\gamma_{j}\int_{0}^{t}dt^{\prime }\;e^{-i\left(\Omega -\omega_{j}\right)t^{\prime }}C_{0}\left(t^{\prime }\right). \end{array}$$

In the rest of the paper, we integrate these equations numerically for the given set of couplings and frequencies.

## 4. Results on the Qubit + Resonator Environment Dynamics

**Figure 2:**
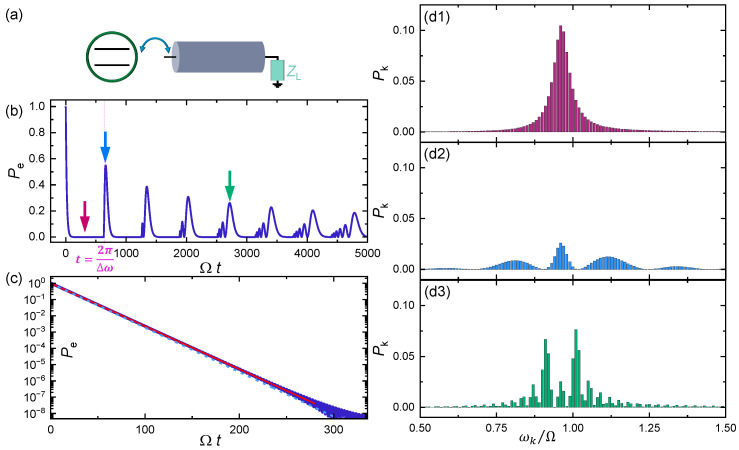
A qubit coupled to a linear Josephson junction array or a transmission line. (**a**) A schematic presentation of the circuit. (**b**) Time-dependent population $p_{e}\left(t\right)$ of the qubit after initialization to the excited state. The transmission line is assumed to be initially in the ground state. The coupling parameter between the qubit and the line is g=0.001. We have chosen Δω=0.01Ω, typically corresponding to either $N=10^{4}$–$10^{5}$ junctions or a 1 m long transmission line, close to that in Ref. [28]. The value of the impedance $Z_{L}$ has almost no effect on $p_{e}\left(t\right)$. (**c**) Initially the qubit decays exponentially, until at t=2π/Δω the first revival sets abruptly in. The solid line is an exponential fit in this range. The dashed line, also following closely the numerical result, is given by the analytic expression with a decay rate $\Gamma =2\pi \frac{g^{2}}{\hbar^{2}}\frac{\Omega }{\Delta \omega^{2}}$, corresponding to a continuum approximation of frequencies. (**d1**–**d3**) Populations of the states in the multimode resonator at three time instants indicated by arrows in (**b**).

Returning first to a Josephson junction array or a finite transmission line, we may write the (angular) frequencies of the multimode resonator as $\omega_{k}=k\Delta \omega$ (exactly for an LC transmission line and approximately for the array well below $\omega_{p}$, see Equation (2)), where the spacing Δω is given by the length of the line or array as discussed above for the latter. Furthermore, we assume the standard coupling as $\gamma_{k}=g\sqrt{k}$, where *g* is the coupling constant arising, e.g., from the capacitance between the qubit and the resonator [28]. This model, with the system depicted in Figure 2a, demonstrates in the absence of true dissipative elements almost periodic exchange of energy between the qubit and the cavity shown in Figure 2b, where the excited state population of the qubit $p_{e}\equiv {\left|C_{0}\right|}^{2}$ is depicted against the normalized time Ωt. This is in contrast to the exponential decay in the case of a resistor as environment. In this numerical example, we chose Δω=0.01 Ω and included N=300 states in the calculation. This energy spacing approximately mimics the experiment of Ref. [28]. We can see that the revivals are not full, and the energy of the qubit is distributed over many states with energies in the neighborhood of ℏΩ. Zooming in to the short time regime as in Figure 2c, we observe the exponential decay of the population over eight orders of magnitude. A closer analysis of the dynamics yields that the decay in short times is indeed exponential, with a decay rate $\Gamma =2\pi \frac{g^{2}}{\hbar^{2}}\frac{\Omega }{\Delta \omega^{2}}$, following the numerical result of Figure 2c. The other important feature in the dynamics, naturally, is the periodic recoveries of $p_{e}\left(t\right)$. The first repopulation demonstrates a sharp peak that sets abruptly on at time t=2π/Δω. We may associate this with the time of flight of a photon with frequency Ω through the transmission line and reflected back; thus, *t* is proportional to the length of the line or *N* in the array. In practical circuits, this recovery time falls into a very short nanosecond regime, meaning that the transmission line acts as a bath only for times shorter than this. In Ref. [28], similar results as in Figure 2b were obtained using the input–output theory [32,33]. The results are robust against different terminations of the line. We also tested the dynamics using different initial states of the system, which did not lead to noticeable changes in the recovery time.

## 5. Heat Bath Formed of Two-Level Systems (Qubits)

As is well known, a set of reactive elements can, however, effectively approximate a dissipative element in the spirit of Caldeira and Leggett [29]. We will next discuss the conditions of forming a heat bath in a solid-state quantum context without actual dissipative building blocks. In particular, we focus on a collection of coupled quantum two-level systems (TLSs), which can, in practice, be formed of Josephson junction based qubits [34] or of unknown structural defects in superconducting circuits [35,36]. A set of harmonic oscillators in the form of superconducting cavities would provide an alternative realization of a Caldeira–Leggett environment. Here, we focus on TLSs. Returning to the archetypal setup, where a central qubit couples to an ensemble of these TLSs, we observe the dynamics of this qubit when initially set to its excited state. We use the same model as above but now with different distributions of energies and couplings of the TLSs. For the sake of clarity of the argument, all the TLSs are again set initially to their ground state, mimicking a zero temperature environment. As we have shown in another context [31], a broad distribution of energies of the TLSs secures the exponential decay of the qubit population in time. This can be seen also analytically, for instance, by standard means re-summing in all orders of perturbation, assuming a large number of uniformly distributed TLS energies. The distribution of energies and couplings of the TLSs is an essential condition for absorbing the energy of the qubit to this bath without recoveries over any practical timescales. In this case, the qubit decays exponentially as

(7) $$|C_{0}{\left(t\right)|}^{2}≃e^{-\Gamma_{0}t}.$$

Here, $\Gamma_{0}=2\pi \nu_{0}\Lambda_{0}^{2}/N$, with $\nu_{0}$, the density of TLSs around Ω, and $\Lambda_{0}^{2}=\sum_{i=1}^{N}\gamma_{i}^{2}/\hbar^{2}$.

In general, for any distribution of energies and couplings, we find that the qubit amplitude $C_{0}\left(t\right)$ in the excited state is governed by the integro-differential equation

(8) $$\begin{array}{ccc} & & {\ddot{C}}_{0}\left(t\right)+\Lambda_{0}^{2}\;C_{0}\left(t\right)=\\ & & -\frac{i}{\hbar^{2}}\sum_{k=1}^{N}\gamma_{k}^{2}\left(\Omega -\omega_{k}\right)\int_{0}^{t}dt^{\prime }e^{i\left(\Omega -\omega_{k}\right)\left(t-t^{\prime }\right)}C_{0}\left(t^{\prime }\right). \end{array}$$

We see immediately that, for the case where all the TLSs have the same energy as the qubit, $\omega_{k}\equiv \Omega$ for all *k*, and the qubit does not decay, even when the couplings $\gamma_{i}$ are fully random; however, it oscillates with the population $|C_{0}{\left(t\right)|}^{2}=\cos^{2}\left(\Lambda_{0}t\right)$; i.e., the Poincare recovery time is $\pi /\Lambda_{0}$.

We can generalize the conclusion above for a bath where $\omega_{k}=\left(1-r\right)\Omega$ for arbitrary positive *r*, meaning detuned equal-energy TLSs in the environment. In this case, Equation (8) leads to $\ddot{D}\left(t\right)-ir\Omega \dot{D}\left(t\right)+\Lambda_{0}^{2}D\left(t\right)=0$, where $D\left(t\right)={\dot{C}}_{0}\left(t\right)$. $C_{0}\left(t\right)$ satisfies the initial conditions $C_{0}\left(0\right)=1$, ${\dot{C}}_{0}\left(0\right)=0$ and ${\ddot{C}}_{0}\left(0\right)=-\Lambda_{0}^{2}$. We then have the oscillatory solution

(9) $$|C_{0}{\left(t\right)|}^{2}=1-\frac{\Lambda_{0}^{2}}{\Lambda_{0}^{2}+{\left(r\Omega /2\right)}^{2}}\sin^{2}\left(\sqrt{\Lambda_{0}^{2}+{\left(r\Omega /2\right)}^{2}}\;t\right).$$

**Figure 3:**
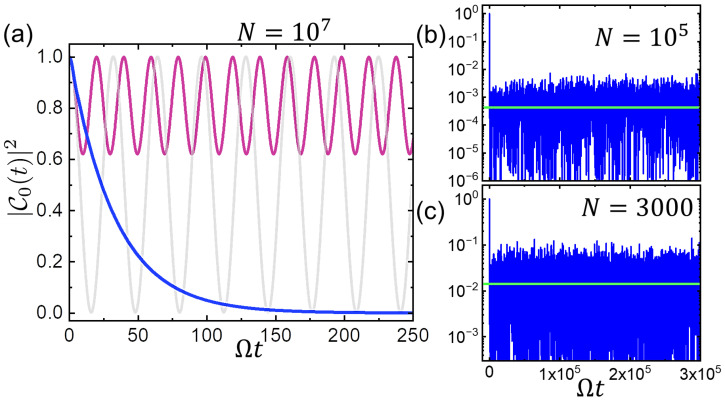
A qubit coupled to a reservoir of $N=10^{7}$ two-level systems in (**a**). The central qubit is coupled to each TLS via coupling constants $\gamma_{i}$ that have a uniform distribution between 0 and its maximum level, corresponding to the overall relaxation rate $\Gamma_{0}=0.03$. The dark blue line corresponds to the evolution of $p_{e}\equiv {\left|C_{0}\right|}^{2}$ in the environment of TLSs with uniform distribution of energies in the range $0<\omega_{i}<2\Omega$ leading to nearly exponential decay. The oscillatory qubit populations of the other curves correspond to uniform environments with $\omega_{i}=\left(1-r\right)\Omega$ for all *i*, with r=0,0.25 for grey and red lines, respectively. These dynamics follow that given by Equation (9) quantitatively. (**b**,**c**) show the population in a similar distributed bath of $N=10^{5}$ and N=3000 TLSs, respectively, over a time period of $\Omega t=3\times 10^{5}$. The horizontal lines are the analytical long time predictions given in the text.

Figure 3a shows the numerically calculated results of $p_{e}\left(t\right)$ for $N=10^{7}$ TLSs and for different choices of parameters following closely the analytical results given above. For a uniform distribution of TLS energies in the range [0,2ℏΩ], the decay is exponential as described above, whereas for TLSs with identical energies, there are periodic revivals in quantitative agreement with the analytic result. These results serve as a warning sign for models where bath spins are assumed to have equal energies. In Figure 3b,c, we numerically monitor the long time behavior of $p_{e}\left(t\right)$ under the same conditions as in the main frame but with $N=10^{5}$ and N=3000 TLSs with distributed energies and couplings. We see that there are no revivals over this long period of time in both cases, and the long time population follows closely the prediction $p_{e}\left(t\to \infty \right)=4\Omega /\left(N\pi \Gamma_{0}\right)$ indicated by the horizontal lines [37]. This result emphasizes the importance of randomness (in couplings and frequencies) to prolong the Poincare recurrence time.

Two possible realizations of such reactive baths can be immediately envisioned. The one that corresponds to our analysis here is that of a qubit coupled to a TLS environment with variable energies: with modern qubits as TLSs, the couplings and energies can be varied almost arbitrarily [34]. One can envison to couple hundreds, perhaps even thousands, of such artificial TLSs to a qubit. A simpler choice could be an ensemble of superconducting resonators with the same idea: here, the tunability is more limited, and instead of TLSs, these resonators work as harmonic oscillators.

## 6. Summary

In summary, it is possible to form a thermal bath on a chip, avoiding recurrences [38] over any practical time scale, in the spirit of Caldeira and Leggett [29] using just reactive elements. However, a one-dimensional array of Josephson junctions or alternatively a transmission line exhibits periodic recoveries on nanosecond time scales in practical physical circuits for two reasons: first, the energy distribution is not dense, and, equally importantly the coupling is not random but essentially equal ($\propto \sqrt{i}$) to each state *i*. Such an environment is thus a heat bath only if it has significant intrinsic dissipation, valid typically for $N>10^{5}$ [18,20], or if it is terminated by a resistive element [39]; in this case the termination itself is the bath. A way around to achieve a true bath is to form a network of harmonic oscillators or TLSs with distributed parameters and couple it to the quantum system.

## Data Availability

Data are contained within the article.
